# SARS-CoV-2 and Europe: timing of containment measures for outbreak control

**DOI:** 10.1007/s15010-020-01420-9

**Published:** 2020-04-09

**Authors:** Chenyu Li, Paola Romagnani, Albrecht von Brunn, Hans-Joachim Anders

**Affiliations:** 1 Medizinische Klinik Und Poliklinik IV, Klinikum Der Universität, 80336 Munich, Germany; 2 Excellence Centre for Research, University of Florence, Florence, Italy; 3 Max Von Pettenkofer-Institute, LMU Munich and German Center for Infection Research (DZIF), Munich, Germany

**Fig. 1:**
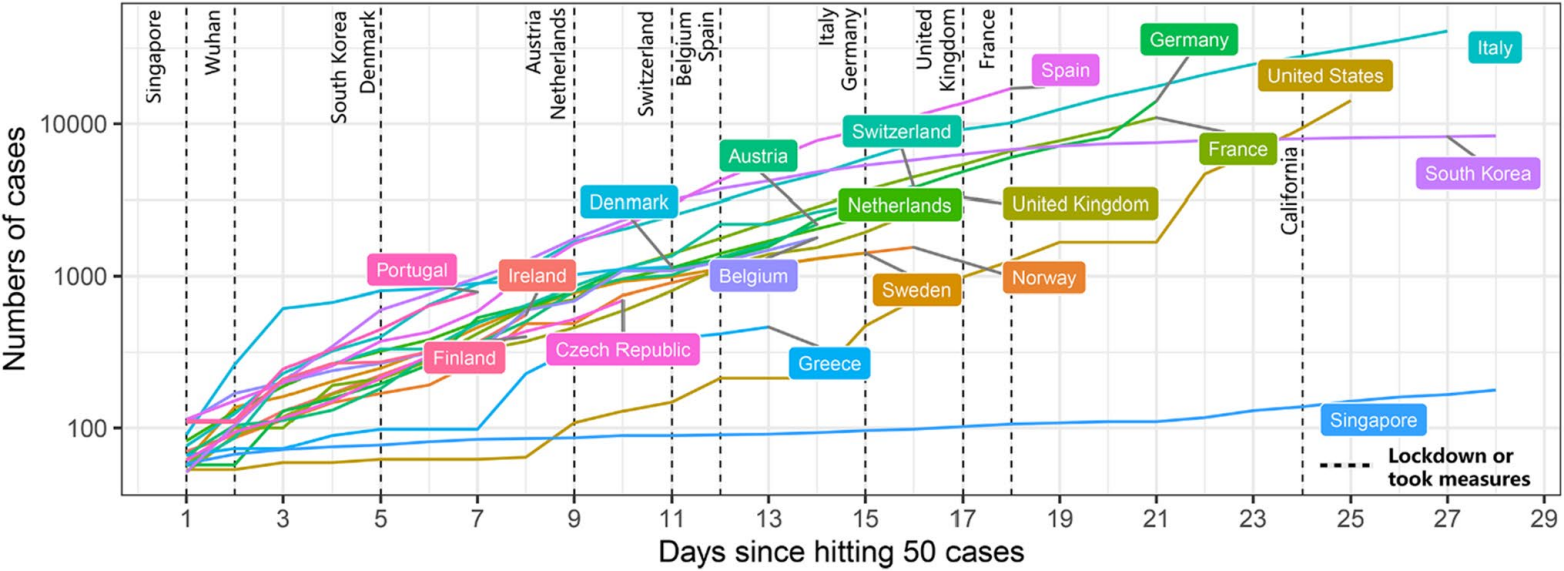
The normalized cumulative curve for the number of cases infected with SARS-CoV-2. The logarithmic scale presents the exponential trend in an apparently linear fashion. The dotted lines represent the days after the outbreak when restrictive containment measures had been installed by the national authorities. Data source: https://www.who.int/emergencies/diseases/novel-coronavirus-2019/situation-reports

**Fig. 2:**
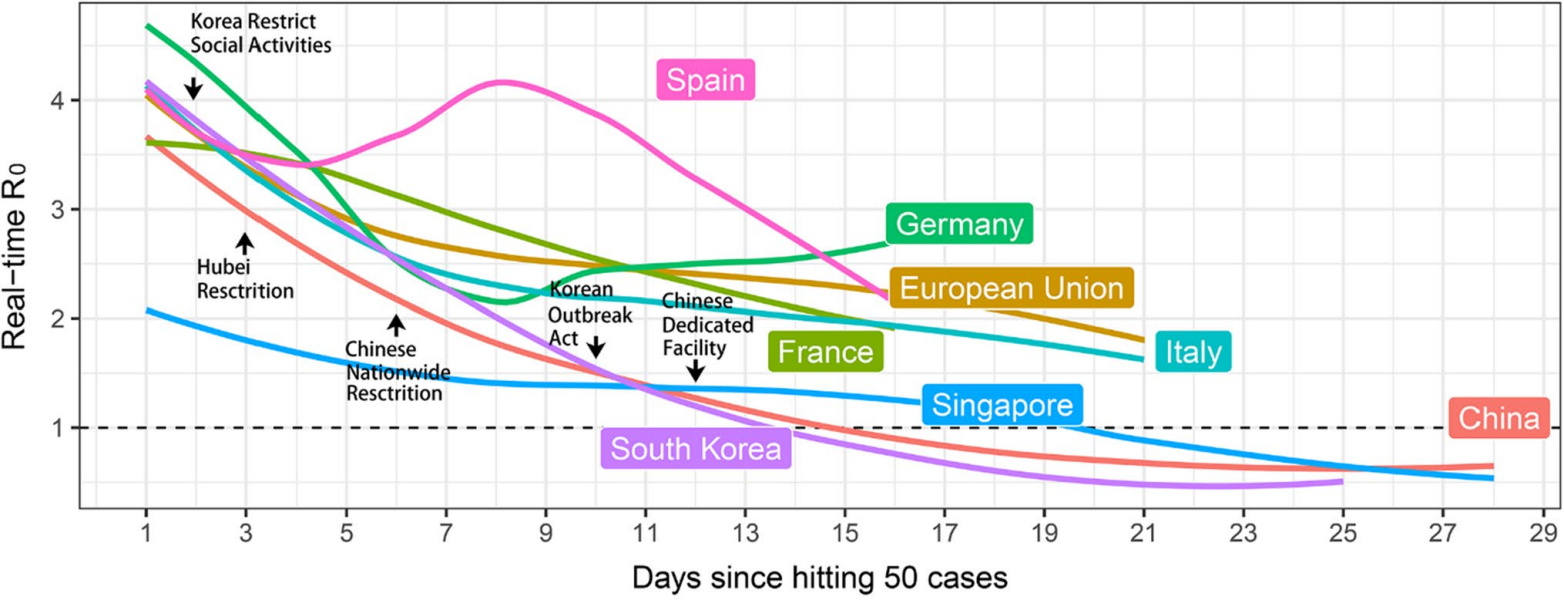
Real-time basic reproduction number (*R*_0_) curve for European Union, Italy, Singapore, South Korea, and China. If *R*_0_ *I* < 1, each existing infection causes less than one new infection and the disease will decline. If *R*_0_ = 1, each existing infection causes one new infection. The disease will stay alive and stable, but there will not be an outbreak or an epidemic. If *R*_0_ > 1, each existing infection causes more than one new infection, and there may be an outbreak or epidemic. *R*_0_ was calculated by using *EpiEstim* package based on a serial interval of 4.4 days. Data source: https://www.who.int/emergencies/diseases/novel-coronavirus-2019/situation-reports

To the editor,

Severe acute respiratory syndrome coronavirus-2 (SARS-CoV-2) infection has reached pandemic proportions, because most infected individuals remain asymptomatic and undocumented [1], which facilitates the rapid dissemination of the virus across borders [2]. Within few weeks, SARS-CoV-2 has infected hundred thousands people in over 200 countries, albeit the numbers increase at a different pace in certain countries (Fig. 1). Singapore, like other Asian countries, installed effective containment measures early, which turned out effective in keeping case numbers low from the start (Singapore) or helped to escape the exponential increase in new infections at some point, e.g. in South Korea and Wuhan, China (Fig. 1) [3, 4]. For the uncertainties about the pathogenicity of SARS-Cov-2 compared to influenza virus Europe did not install strict containment measures early, so that since February 21, the numbers of infected people closely keep following an exponential trend (*R*_0_ = 2–3, Figs. 1 and 2) [5]. Italy has been most affected and only gradually installed nation-wide containment measures up to a complete lockdown on March 9. Meanwhile, the rest of Europe kept hesitating and started to install drastic containment measures not before March 15 so the basic reproduction number (*R*_0_) has remained > 2 (Fig. 2). A persistent *R*_0_ of > 2 implies that each infected individual keeps infecting more than two others, i.e. an ongoing exponential increase in infection numbers enforcing the epidemic, with all its consequences on healthcare system overload and mortality.

**Fig. 3:**
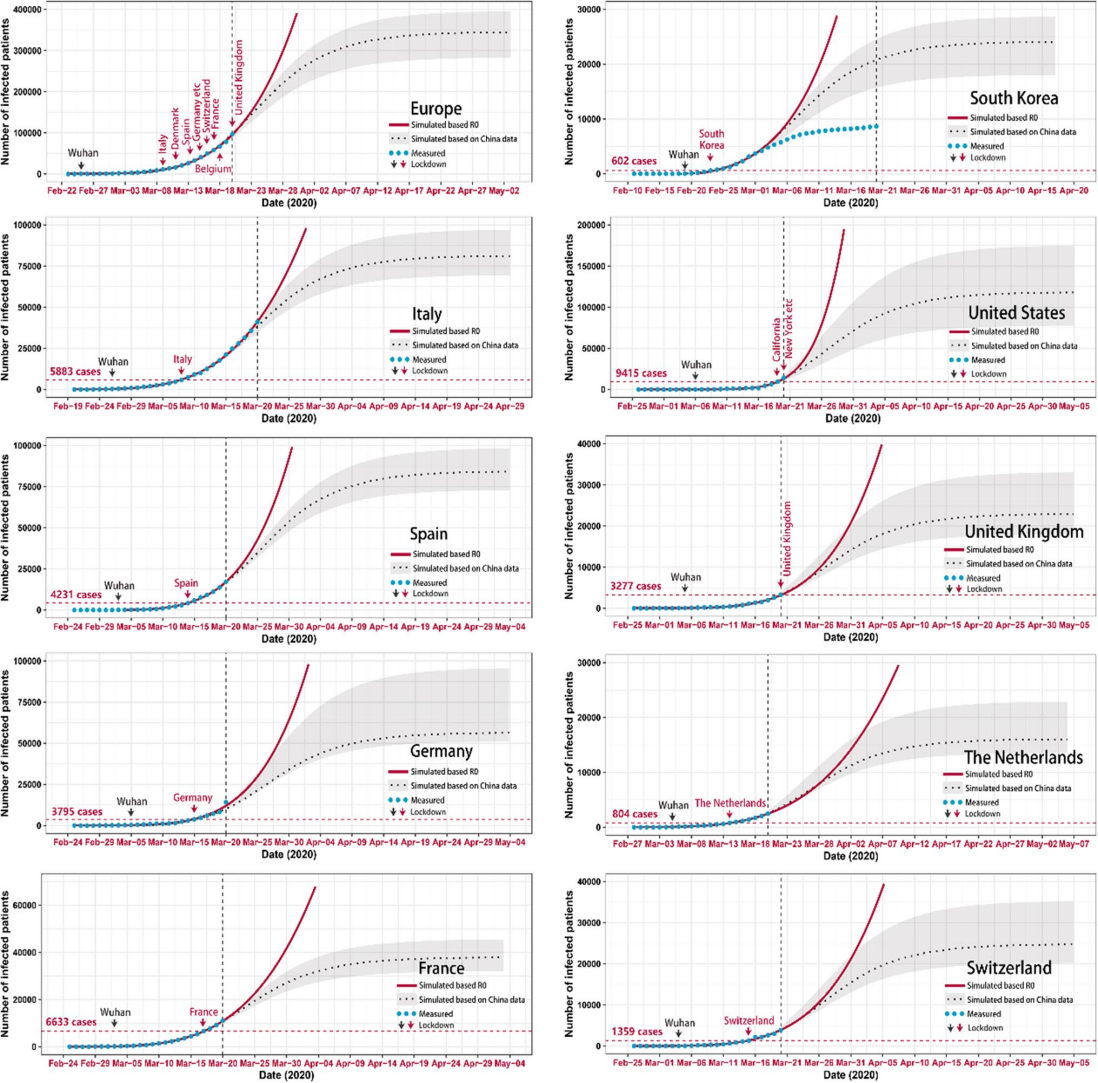
Fitting of the cumulative curve of measured infected patients in the European countries and South Korea based on historical Chinese data. The red line represents the exponential curve estimated based on the last basic reproduction number. The blue dotted line represents the reported cases starting from the time-point of each national outbreak as indicated in the red *x*-axis for each country, respectively. The black dotted line represents cumulative curve of number of infected patients based on historical Chinese data with the gray area for 1 to 3 days incubation periods. A black arrow indicates the relative time point when Wuhan/China installed restrictive containment measures to illustrate the respective delay for each country. The horizontal red line indicates the number of SARS-CoV-2 positive cases at the time of national lockdown. The curves were estimated based on basic reproduction number which calculated by using *EpiEstim* package with a serial interval of 4.4 days. Data source: https://www.who.int/emergencies/diseases/novel-coronavirus-2019/situation-reports

It has been proposed by comparing the trend of the Italian outbreak with that of the Hubei province in China, that case numbers would start to deviate from the exponential increase around March 11 [6]. However, this assumption ignored that Italy`s lockdown was installed 13 days after the one of Hubei, when normalizing for the time when hitting 50 cases (Fig. 1). Indeed, judging from the numbers reported up to March 21 the Italian numbers of infected cases keep following the exponential trend (Fig. 3).

Assuming that the Italian trend does not follow the Chinese experience because drastic containment measures had been taken much later in the course of the national epidemic, the question arises what we have to expect of the other European countries and the United States depending on their respective time delay in deciding for a national lockdown to limit the outbreak? Assuming that the definition of full nationwide lockdown was reached in Denmark on March 11, in Germany, The Netherlands, France, Spain, Austria, Ireland, Poland, and Czech Republic on March 15, and in Switzerland on March 16, the different delays in the starting a complete lockdown compared to Wuhan/Hubei, China and what happens in Italy are shown in Fig. 1. We would predict a longer delay will translate into a later deviation from the exponential increase in cases and a higher number of cases altogether for the respective country (Fig. 3).

Assuming that the time point of installing drastic containment measures is a central determinant of when a country will deviate from the exponential increase in numbers of infected people, the authors advocate an immediate lockdown to all countries that have not done so to limit any further transmission and exponential spreading of SARS-CoV-2 and its devastating consequences. Beyond physical distancing, generous testing to identify and isolate individuals with asymptomatic SAS-Cov-2 infections, are equally important to control the pandemic.

Chenyu Li, Albrecht von Brunn, Hans-Joachim Anders, University of Munich, LMU, Germany.

Paola Romagnani, University of Florence, Italy.
